# An integrated in vitro and in silico approach to assess the potential inhibitory actions of auxins on human placental glutathione S-transferase P1-1

**DOI:** 10.1007/s00210-026-05085-3

**Published:** 2026-02-16

**Authors:** Pavel Awat Husamadin, Ozlem Dalmizrak, Kerem Teralı, Nazmi Ozer

**Affiliations:** 1 Department of Biomedical Science, Komar University of Science and Technology, Sulaimaniyah, Iraq; 2 Department of Medical Biochemistry, Faculty of Medicine, Near East University, Nicosia, TRNC, Mersin 10 99138 Turkey; 3 Department of Medical Biochemistry, Faculty of Medicine, Cyprus Health and Social Sciences University, Kutlu Adalı Boulevard, Morphou, TRNC, Mersin 10, 99750 Turkey; 4 Department of Biochemistry, Faculty of Pharmacy, Girne American University, Kyrenia, TRNC, Mersin 10 99428 Turkey

**Keywords:** Human placental glutathione *S*-transferase P1-1, Indoleacetic acid, Indolepropionic acid, Indolebutyric acid, Competitive inhibition

## Abstract

Glutathione *S*-transferases (GSTs) are multifunctional enzymes involved in the metabolism of a wide variety of xenobiotics and endogenous compounds. Since some GST isozymes are overexpressed in a variety of malignancies and have been shown to play roles in the development of drug resistance, GSTs have become a promising therapeutic target. Although numerous natural and plant-derived compounds have been evaluated as GST inhibitors, the interaction between human GSTP1-1 and auxins-indole-derived plant hormones that are also detectable in human biological systems-has not been previously characterized. The present study aimed to investigate whether selected auxins can modulate the activity of human placental GSTP1-1 (*hp*GSTP1-1) and to elucidate the mechanistic and structural basis of any interactions. The inhibitory effects of indole-3-acetic acid (IAA), indole-3-propionic acid (IPA), and indole-3-butyric acid (IBA) on *hp*GSTP1-1 were evaluated using in vitro enzyme kinetic assays. *IC*_50_ determination was conducted at various auxin concentrations. Subsequently, through inhibitory kinetic studies, the inhibition types and kinetic parameters were determined. In parallel, molecular docking was performed to provide structural insight into interactions between *hp*GSTP1-1 and auxins. All three auxins inhibited *hp*GSTP1-1 activity in a concentration-dependent manner, with *IC*_50_ values of 7.9 mM (IAA), 6.5 mM (IPA), and 4.2 mM (IBA). Kinetic analyses revealed competitive inhibition with respect to both substrates. The *K*_i_ values from statistical analysis/secondary plots for IAA, IPA, and IBA at [CDNB]_f_-[GSH]_v_ and at [GSH]_f_-[CDNB]_v_ were 4.00 ± 0.62 mM/2.38 mM; 3.33 ± 0.23 mM/2.84 mM; 3.33 ± 0.22 mM/2.61 mM and 3.30 ± 0.24 mM/2.38 mM; 3.52 ± 0.24 mM/2.84 mM; 2.14 ± 0.16 mM/2.61 mM, respectively. These inhibitory auxins were thought to be held mainly by the hydrophilic amino acid residues that are located at the glutathione-binding site of the enzyme. This study provides a biochemical and structural characterization of weak, competitive inhibition of *hp*GSTP1-1 by auxins based on combined in vitro and in silico analyses. While the inhibitory potency is limited and unlikely to be pharmacologically relevant at physiological concentrations, the combined in vitro and in silico findings offer valuable mechanistic and structural insight into auxin–GSTP1-1 interactions.

## Introduction

Glutathione *S*-transferases (GSTs; EC 2.5.1.18) constitute a large family of eukaryotic and prokaryotic metabolic isoenzymes that are profoundly known to be responsible for phase II detoxification by catalyzing the conjugation of xenobiotics to reduced glutathione (GSH), a low molecular weight thiol tripeptide that serves as the nucleophilic cosubstrate in GST-mediated reactions, thereby neutralizing toxic compounds and facilitating their elimination from the body via bile or urine (Coles and Ketterer 1990; Sheehan et al. 2001; Alnasser 2025). In addition to their classical detoxification function, GSTs participate in several other cellular processes, including steroid and leukotriene biosynthesis, peroxide degradation, double bond isomerization, dehydroascorbate reduction, Michael addition, and non-catalytic “ligandin” activity (i.e., ligand binding and transport) (Hayes et al. 2005). Based on their structural, biological, and immunological properties, GSTs are divided into three major superfamilies: cytosolic, mitochondrial (kappa-class), and microsomal GSTs, the latter also known as membrane-associated proteins in eicosanoid and glutathione metabolism (MAPEGs) (Hayes et al. 2005; Aloke et al. 2024). Cytosolic GSTs represent the largest group and are further classified into alpha, beta, delta, epsilon, zeta, theta, mu, nu, pi, tau, phi, and omega classes. In humans, GST classes alpha, zeta, theta, mu, pi, sigma, and omega are found. Concurrently, the mitochondrial GSTs share a deep evolutionary relationship with the cytosolic GSTs; both cytosolic and mitochondrial GSTs form dimers (Mannervik et al. 2005). However, heterodimers of cytosolic GSTs have been identified containing chains belonging to the same class (Mannervik et al. 1985). The MAPEG family comprises of four subgroups (I–IV), and in humans six MAPEG isoenzymes have been identified that belong to subgroups I, II, and IV. Like the cytosolic and mitochondrial GSTs, several MAPEGs such as MGST1 catalyze the conjugation of GSH to a number of electrophilic compounds (Hayes et al. 2005). Other members additionally catalyze the reactions in leukotriene and prostaglandin biosynthesis (Oakley 2011). One of the important properties of GSTs is that their activities are inducible, albeit not entirely but usually through the metabolized electrophiles (Pickett and Lu 1989). GST function, therefore, is one of the important factors in protecting cells from acute toxic chemical assaults. In addition, GSTs can also be protective against cancer since cancer cell formation requires the covalent modification of DNA by electrophiles derived from carcinogens or clastogens, which are detoxified by GSTs (Whalen and Boyer 1998). However, it has been reported that in several cases, elevated levels of GSTs are involved in drug resistance. There is growing evidence suggesting that GST isoenzymes are capable of having different roles in the body. Apart from their catalytic role in protecting cells against xenobiotics, GST isoenzymes can function in the detoxification of chemotherapeutic agents, initiating the development of drug resistance through the inactivation of the anticancer compounds via GSH conjugation (Hayes and Pulford 1995; Dirven et al. 1996). GSTP1-1 has attacted considerable attention in cancer biology because it is frequently overexpressed in a variety of human tumors (Black et al. 1990; Bai et al. 1996; O’Brien et al. 2000; Hawale et al. 2025). This indicates that GST isoenzymes are capable of forming chemotherapeutic drug resistance in tumors (Townsend and Tew 2003).

Since chemotherapy provides the most effective treatment method for cancer, resistance to anticancer chemotherapy can be a serious obstacle in treating cancer. Primary and acquired resistance of tumor cells to antineoplastic drugs can be a serious cause of the limited efficiency of chemotherapy. Potentially, tumors can be intrinsically drug resistant or develop resistance during the treatment. This is a phenomenon that is known as multidrug resistance (MDR). The problem with acquired resistance is that tumors not only become resistant to the drugs originally used in treatment, but also become cross-resistant to other drugs. Therefore, it is in this context that inhibiting the activity of GSTs has the potential to be used as a therapeutic strategy to reverse MDR (Ban et al. 1996; Tew et al. 1997; Yu et al. 2009; Chen et al. 2013).

Auxins are indole-derived plant hormones that play essential roles in regulating plant growth and development (Davies 1995; Woodward and Bartel 2005). The most common naturally occurring auxins include indoleacetic acid (IAA), indolepropionic acid (IPA), and indolebutyric acid (IBA), all of which share an indole ring and a carboxylic acid moiety (Simon and Petrasek 2011). Auxin research is considered one of the oldest fields in plant biology. Early experimental work on auxin was conducted by Charles Darwin, who investigated the effects of hypothetical signaling substances regulating plant shoot elongation and thereby facilitating tropic growth toward light (Darwin 1880). Although auxins are primarily studied in plant biology, they are not foreign to human physiology. IAA, in particular, can be produced endogenously using tryptophan metabolism either through the tryptamine pathway or the indole-3-pyruvic acid pathway (Liu et al. 2002). When IAA is synthesized from tryptophan, it can be detectable in urine, blood plasma, and even in the central nervous system. It has been also stated that IAA can be produced by the liver, hippocampus, kidney, cerebrospinal fluid, and the midbrain (Kim et al. 2020). Moreover, patients with neuromuscular diseases, phenylketonuria, diabetes mellitus, hereditary syndromes with symptoms of mental deterioration, intermittent cerebellar ataxia, liver injury, and cancer can produce very high amounts of IAA endogenously (Kim et al. 2020). Moreover, humans are routinely exposed to auxins through dietary intake (Lopez-Bucio et al. 2026).

Previous studies demonstrated that auxins can interact with GSTs in plants, involving in cellular detoxification of noxious compounds and protection against oxidative stress (Hasanuzzaman et al. 2020). The implications of GSTs in the detoxification of herbicides (Timmerman 1989) and in defending tissues against infectious microorganisms (Levine et al. 1994) have been studied and reported. Intriguingly, previous research has shown that plant GST5 activity is inhibited by 55% with an *IC*_50_ value of 1.3 mM in the presence of 2 mM IAA (Watahiki et al. 1995).

While most research on auxin–glutathione S-transferase (GST) interactions has focused on plant systems, there is very limited direct evidence that plant auxins or indole-3-acetic acid (IAA) interact with human GST enzymes under physiological conditions. An older biochemical study demonstrated that a photooxidation product of the plant auxin IAA, 3-methyleneoxindole, can function as an affinity label for pi-class GST and inactivate it, suggesting that certain indole derivatives can engage mammalian GST active sites under experimental conditions; this compound preferentially reacted with tryptophan residues in the pig GSTP1-1 active site region, implying potential non-physiological binding interactions between indole-derived compounds and GSTs in mammals (Pettigrew et al. 2001). In humans, GSTP1-1 is known to be inhibited by various plant-derived molecules such as polyphenols (Guneidy et al. 2022; Stoian et al. 2025), but specific auxin–GST interactions in mammalian systems are not established in the literature**.** This implies that, although certain indole-related products of auxin metabolism or oxidation may interact with human GSTs in controlled biochemical assays, there is no clear evidence that canonical plant auxins act as physiological ligands or regulators of human GSTP1-1. In this context, the present study aimed to evaluate the inhibitory effects of IAA, IPA, and IBA on *hp*GSTP1-1 activity using an integrated in vitro and in silico approach. Enzyme inhibition was characterized through half-maximal inhibitory concentration (*IC*_50_) determination and detailed kinetic analyses, while molecular docking was employed to gain structural insight into the binding interaction between *hp*GSTP1-1 and the tested auxins.

## Materials and methods

### Chemicals

Human placental glutathione *S*-transferase P1-1 (*hp*GSTP1-1), l-glutathione reduced (GSH), 1-chloro-2,4-dinitrobenzene (CDNB), sodium azide, bovine serum albumin (BSA), ethylenediaminetetraacetic acid disodium salt dihydrate (EDTA), sodium phosphate monobasic, sodium phosphate dibasic dodecahydrate, 3-indoleacetic acid (IAA), 3-indolepropionic acid (IPA), indolebutyric acid (IBA) were purchased from Sigma-Aldrich, Inc. (St. Louis, MO, USA). The *hp*GSTP1-1 enzyme (Product No: G8642) was supplied as a lyophilized powder with a specific activity of 25–125 U/mg protein. For the lot (123K37791) used in this study, protein content determined by the biuret method was 69% (minimum specification ≥ 25%), and the enzymatic activity was 70 units/mg protein.

### Preparation of the stock enzyme, substrate, and inhibitor solutions

The *hp*GSTP1-1 enzyme stock was prepared by dissolving 2 mg of lyophilized enzyme in 1 mL of 100 mM sodium phosphate buffer, pH 6.5, containing 1 mM EDTA. Aliquots of the enzyme stock solution were stored at − 20 °C until use. For *IC*_50_ determination and kinetic experiments, the enzyme stock (2 mg/mL) was diluted 20-fold or 35-fold with 100 mM sodium phosphate buffer, pH 6.5, containing 1 mM EDTA and 0.05% (w/v) BSA, respectively. Stock solutions of GSH were prepared in filtered distilled water, whereas CDNB was dissolved in ethanol. IAA and IPA were dissolved in ethanol, while IBA was dissolved in filtered distilled water. The final ethanol concentration in the reaction mixture did not exceed 5% (v/v) to minimize the solvent-induced inhibition of *hp*GSTP1-1.

### Determination of the *hp*GSTP1−1 enzyme activity

*Hp*GSTP1-1 activity was measured according to the method of Habig and Jakoby, with minor modifications (Habig and Jakoby 1981). The activity of *hp*GSTP1-1 was monitored spectrophotometrically by measuring the increase in absorbance at 340 nm resulting from the conjugation of GSH with CDNB to form GS–DNB conjugate. Reactions were initiated by the addition of CDNB, and absorbance changes were recorded for 20 s using a Perkin Elmer LAMBDA 25 UV/VIS Spectrophotometer (Waltham, MA, USA). A parallel non-enzymatic reaction, which contained all constituents of the reaction mixture excluding the *hp*GSTP1-1 enzyme, was included for each assay, and its absorbance change was substracted from the corresponding enzymatic reaction. All measurements were performed at 37 °C and in triplicate. Average activity (U/mL) was calculated using a molar extinction coefficient of 9.6 mM^−1^ cm^−1^for the GS–DNB conjugate. First, U/mL values were converted to specific activity (U/mg protein) values, and the latter were used to depict Lineweaver–Burk and secondary plots (Segel 1975). One unit of the *hp*GSTP1-1 enzyme activity was defined as the amount of the enzyme that catalyzed the formation of 1 μmol GS–DNB per min at pH 6.5 and 37 °C.

### Determination of *IC*_50_ values

The inhibitory effects of IAA, IPA, and IBA on *hp*GSTP1-1 activity were evaluated using inhibitor concentrations ranging from 0.3125 to 5 mM. The reaction mixture (final volume 800 µL) contained 100 mM sodium phosphate, pH 6.5, containing 1 mM EDTA, 1 mM CDNB, 1 mM GSH, and the appropriate amount of the enzyme (Habig and Jakoby 1981). All assays were performed in triplicate at each inhibitor concentration. *IC*_50_ values were determined by plotting the logarithm of the percent remaining activity versus inhibitor concentration (Segel 1975).

### Inhibitory kinetic studies

Inhibitory kinetic studies were performed in the absence and presence of increasing concentrations (0.25, 0.5, 1, and 2 mM) of IAA, IPA, and IBA. Assays were conducted under two conditions (i) fixed CDNB concentration (1 mM) with variable GSH concentrations (0.1, 0.2, 0.4, 0.8, and 1.6 mM) and (ii) fixed GSH concentration (1 mM) with variable CDNB concentrations (0.1, 0.2, 0.4, 0.8, and 1.6 mM). The reaction mixture was composed of 100 mM sodium phosphate buffer, pH 6.5, containing 1 mM EDTA, different concentrations of the inhibitor (IAA, IPA, or IBA), substrates at either 1 mM [CDNB]_f_–[GSH]_v_ or 1 mM [GSH]_f_ –[CDNB]_v_, and the appropriate amount of the enzyme. All of the measurements were taken at 37 °C and in triplicate (Habig and Jakoby 1981). Lineweaver–Burk plots and secondary plots were generated to determine the inhibition type and kinetic parameters (Segel 1975).

### Protein determination

The amount of protein in the stock enzyme solution was determined by the method of Lowry et al. (1951), with bovine serum albumin as the standard.

### Molecular docking

The crystal structure of recombinant human glutathione *S*-transferase P1-1 (GSTP1-1) in complex with 1-(*S*-glutathionyl)−2,4-dinitrobenzene (GS–DNB) (PDB entry: 18GS; resolution: 1.90 Å; *R*_free_: 0.238; *R*_work_: 0.181) (Oakley et al. 1999) was selected for use as input to the JAMDA protein–ligand docking tool (Schellhammer and Rarey, 2007; Henzler et al. 2014; Flachsenberg et al. 2020) available at https://proteins.plus, provided that GSTP1-1 was competitively inhibited by the auxins under study. The three-dimensional conformers of indoleacetate (IAA) (ZINC entry: ZINC83860), indolepropionate (IPA) (ZINC entry: ZINC7700) and indolebutyrate (IBA) (ZINC entry: ZINC57378) were downloaded from the ZINC public access database for virtual screening (Sterling and Irwin, 2015) available at https://zinc15.docking.org/. Auxins were docked onto GSTP1-1, which was prepared for docking by using the Protoss hydrogen prediction tool (Stierand et al. 2006; Lippert and Rarey 2009) available at https://proteins.plus that adds missing hydrogen atoms and establishes an optimal hydrogen-bonding network, in the presence of structurally relevant water molecules. The region of interest on GSTP1-1 was defined by the cocrystallized ligand GS–DNB, with a site radius of 6.5 Å, and protein–ligand docking was executed with medium precision. Favorable noncovalent interactions between GSTP1-1 and auxins were visualized by using the PoseView protein–ligand interaction mapping tool (Fricker et al. 2004; Stierand et al. 2006) available at https://proteins.plus.

### Statistical analysis

The Lineweaver–Burk plots and secondary plots were constructed, and inhibition types and kinetic parameters were estimated using the slopes and intercepts derived from these graphs. Statistical analysis was also carried out to confirm the validity of these preliminary findings. For this purpose, the nonlinear regression module of IBM SPSS Statistics (version 20) was implemented (SPSS Inc., Chicago, IL, USA). Enzyme kinetic parameters (*V*_m_, *K*_m_, and *K*_i_) were estimated by fitting the experimental initial velocity data to appropriate inhibition models using nonlinear regression. This approach directly fits the Michaelis–Menten equation and its inhibition variants to the full dataset, avoiding data transformation and minimizing error propagation.

## Results

### Substrate kinetic studies

The substrate kinetic studies were performed to determine the catalytic properties of *hp*GSTP1-1 under the assay conditions used in this study. Enzyme activity was measured at fixed concentration of CDNB (1 mM) with variable GSH concentrations (0.1, 0.2, 0.4, 0.8, and 1.6 mM) and conversely, at a fixed concentration of GSH (1 mM) with variable CDNB concentrations (0.1, 0.2, 0.4, 0.8, and 1.6 mM). Lineweaver–Burk analysis yielded maximum velocity (*V*_*m*_) values of 263 ± 16 µmol/min-mg protein for the [CDNB]_f_–[GSH]_v_ condition and 233 ± 10 µmol/min-mg protein for the [GSH]_f_–[CDNB]_v_ condition. The Michaelis constants (*K*_*m*_) were 1.53 ± 0.12 mM for GSH and 1.35 ± 0.10 mM for CDNB (Table 1).

**Table 1:** Kinetic parameters for the inhibition of *hp*GSTP1-1 by IAA, IPA and IBA

Kinetic parameters	IAA*	IPA*	IBA*
*V*_m_ [CDNB]_f_-[GSH]_v_ µ mole/min-mg Protein	263 ± 16	263 ± 16	263 ± 16
*V*_m_ [GSH]_f_-[CDNB]_v_ µ mole/min-mg Protein	233 ± 10	233 ± 10	233 ± 10
*K*_m_^GSH^, mM	1.53 ± 0.12	1.53 ± 0.12	1.53 ± 0.12
*K*_m_^CDNB^ mM	1.35 ± 0.10	1.35 ± 0.10	1.35 ± 0.10
*IC*_50_, mM	7.9	6.5	4.2
Inhibition type with GSH	Competitive	Competitive	Competitive
Inhibition type with CDNB	Competitive	Competitive	Competitive
*K*_i_^GSH^, mM (Statistical analysis)	4.00 ± 0.62	3.33 ± 0.23	3.33 ± 0.22
*K*_i_^GSH^, mM (Secondary plots)	2.38 mM	2.84	2.61
*K*_i_^CDNB^, mM (Statistical analysis)	3.30 ± 0.24	3.52 ± 0.24	2.14 ± 0.16
*K*_i_^CDNB^, mM (Seconday plots)	2.38 mM	2.84	2.61

^*^IAA Indoleacetic acid, *IPA Indolepropionic acid, ^*^IBA Indolebutyric acid

### Dose–dependent inhibition of *hp*GSTP1-1 by auxins

**Fig. 1:**
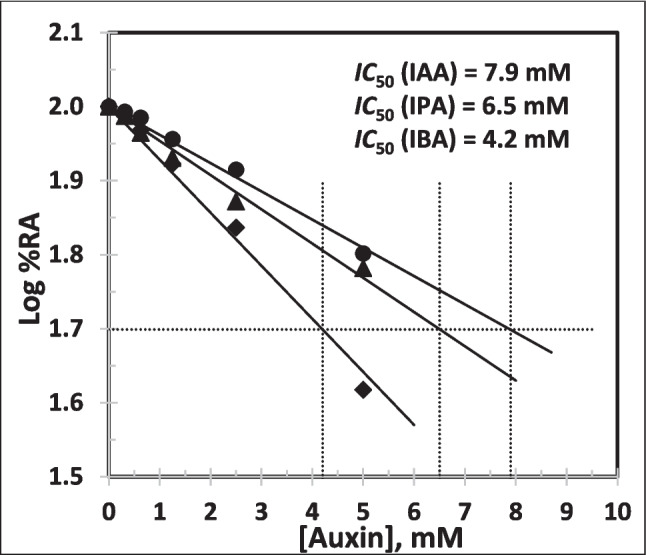
Dose-dependent inhibition of *hp*GSTP1-1 by auxins. (●) IAA, (▲) IPA, (■) IBA

The inhibitory effects of IAA, IPA, and IBA on *hp*GSTP1-1 activity were evaluated by the addition of different inhibitor concentrations (0.3125, 0.625, 1.25, 2.5, and 5 mM) into the reaction mixture. All three auxins produced a concentration-dependent decrease in enzyme activity. Logarithm of percentage remaining activity versus inhibitor concentration plot were used to calculate *IC*_50_ values. The *IC*_50_ values were determined to be of 7.9 mM for IAA, 6.5 mM for IPA, and 4.2 mM for IBA (Fig. 1), indicating that IBA was the most potent inhibitor among the tested auxins.

### Inhibitory kinetic interactions between *hp*GSTP1-1 and IAA

**Fig. 2:**
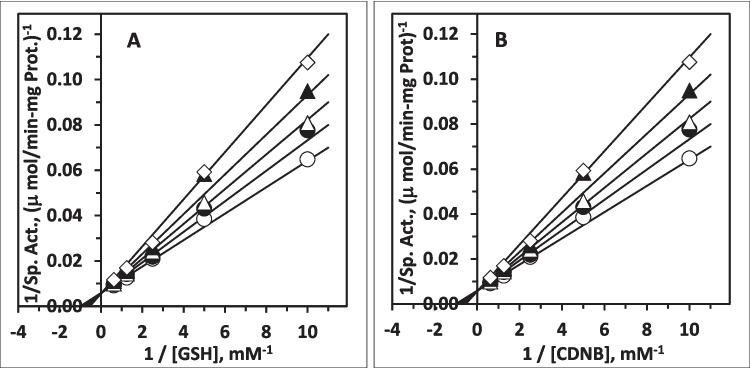
Lineweaver–Burk plots for inhibition of *hp*GSTP1-1 by IAA. **A**, Plots obtained at a fixed [CDNB] (1 mM) and varying [GSH] (0.1–1.6 mM) in the absence and presence of increasing IAA concentrations **B**, Plots obtained at a fixed [GSH] (1 mM) and varying [CDNB] (0.1–1.6 mM) in the absence and presence of increasing IAA concentrations. [IAA]: (○), 0; (●), 0.25; (Δ), 0.5; (▲), 1.0; (□), 2.0 mM

**Fig. 3:**
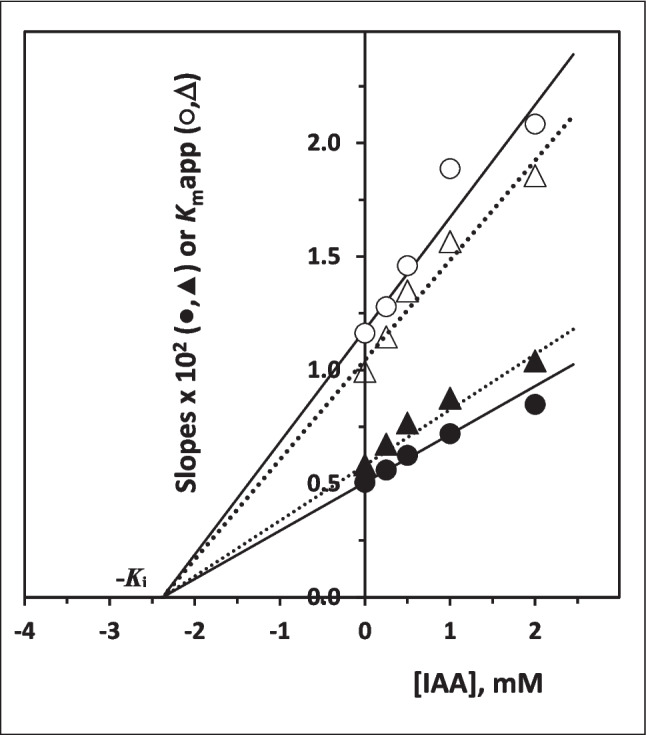
Secondary plots for determination of *K*_i_ values for IAA. Fixed 1 mM [CDNB] and variable [GSH] (solid line) slope (○), and intercept (●); and fixed 1 mM [GSH] and variable [CDNB] (dashed line); slope (Δ), intercept (▲)

To elucidate the mode of inhibition and determine inhibition constants, kinetic analyses were conducted in the presence of increasing concentrations of IAA (0.25, 0.5, 1, and 2 mM). Lineweaver–Burk plots demonstrated straight lines intersecting at y-axis, consistent with the competitive inhibition, with respect to both GSH and CDNB (Fig. 2A, B). Secondary plots and nonlinear regression analysis yielded* K*_i_ values of 4.00 ± 0.62 mM (statistical analysis)/2.38 mM (secondary plots) for GSH, and 3.30 ± 0.24 mM (statistical analysis)/2.38 mM (secondary plots) for CDNB (Fig. 3, Table 1).

### Inhibitory kinetic interactions between *hp*GSTP1-1 and IPA

**Fig. 4:**
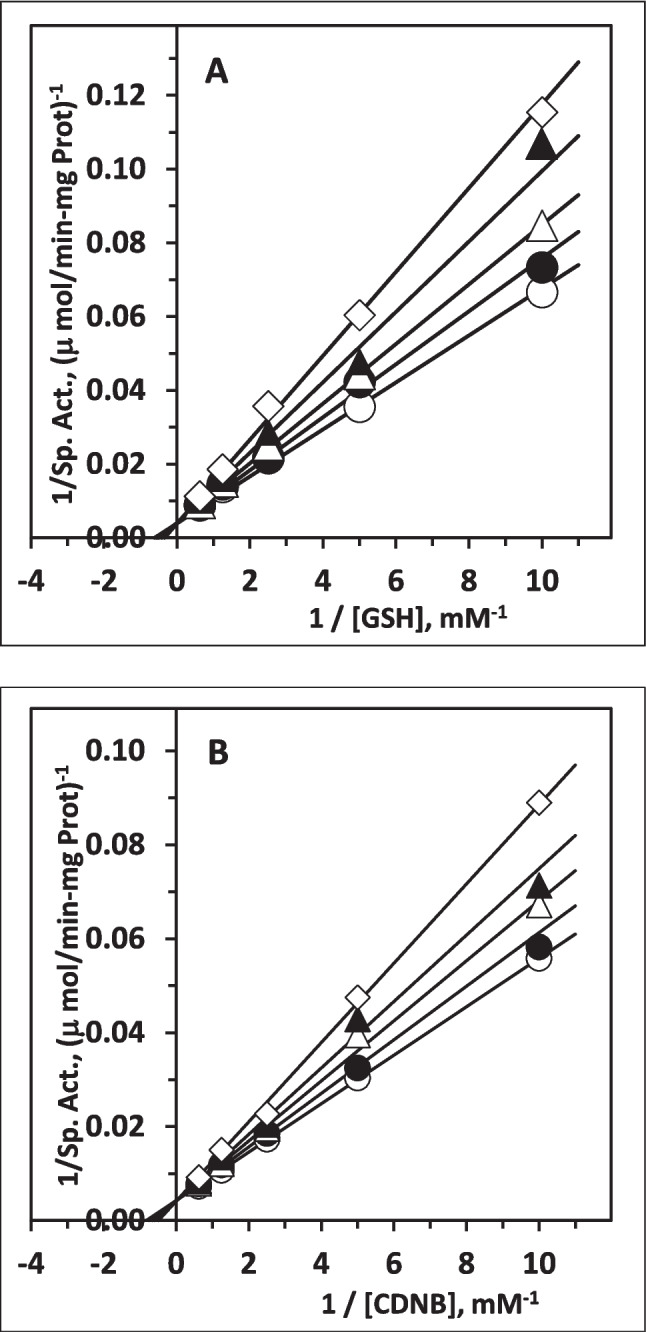
Lineweaver–Burk plots for inhibition of *hp*GSTP1-1 by IPA. **A**, Plots obtained at a fixed [CDNB] (1 mM) and varying [GSH] (0.1–1.6 mM) in the absence and presence of increasing IPA concentrations **B**, Plots obtained at a fixed [GSH] (1 mM) and varying [CDNB] (0.1–1.6 mM) in the absence and presence of increasing IPA concentrations. [IPA]: (○), 0; (●), 0.25; (Δ), 0.5; (▲), 1.0; (□), 2.0 mM

**Fig. 5:**
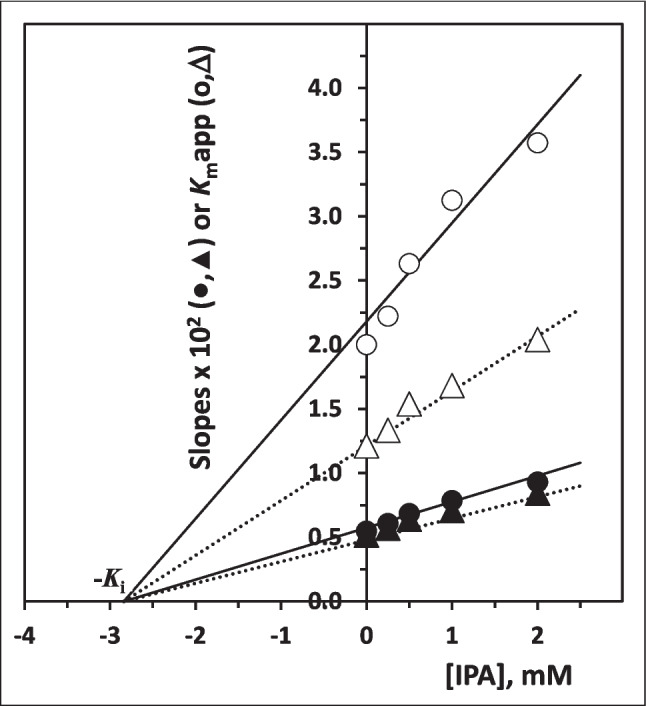
Secondary plots for determination of *K*_i_ values for IPA. Fixed 1 mM [CDNB] and variable [GSH] (solid line) slope (○), and intercept (●); and fixed 1 mM [GSH] and variable [CDNB] (dashed line); slope (Δ), intercept (▲)

The inhibitory behavior of IPA was examined under identical experimental conditions. Lineweaver-Burk plots revealed competitive inhibition with respect to both substrates (Fig. 4A, B). *K*_i_ values derived from nonlinear regression were 3.33 ± 0.23 mM for GSH and 3.52 ± 0.24 mM for CDNB, while corresponding values obtained from secondary plots were 2.84 mM for both substrates (Fig. 5, Table 1).

### Inhibitory kinetic interactions between *hp*GSTP1-1 and IBA

**Fig. 6:**
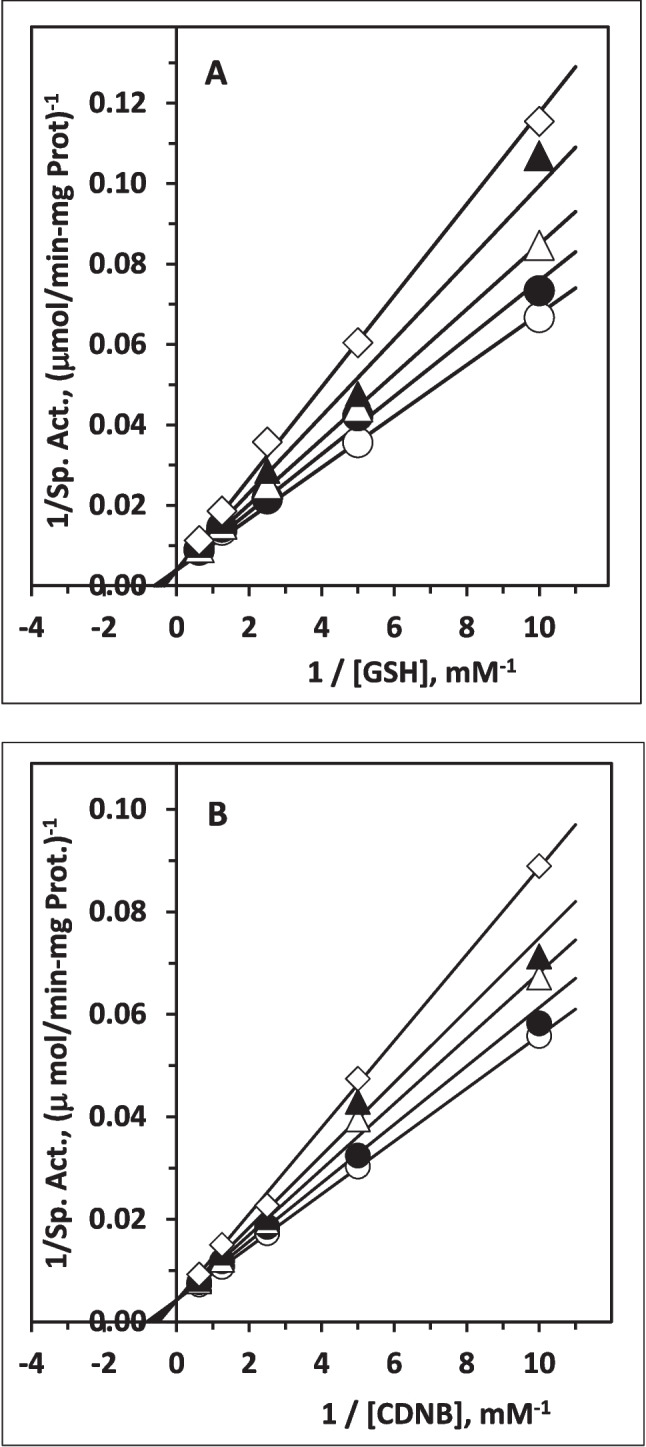
Lineweaver–Burk plots for inhibition of *hp*GSTP1-1 by IBA. **A**, Plots obtained at a fixed [CDNB] (1 mM) and varying [GSH] (0.1–1.6 mM) in the absence and presence of increasing IBA concentrations **B**, Plots obtained at a fixed [GSH] (1 mM) and varying [CDNB] (0.1–1.6 mM) in the absence and presence of increasing IBA concentrations. [IBA]: (○), 0; (●), 0.25; (Δ), 0.5; (▲), 1.0; (□), 2.0 mM

**Fig. 7:**
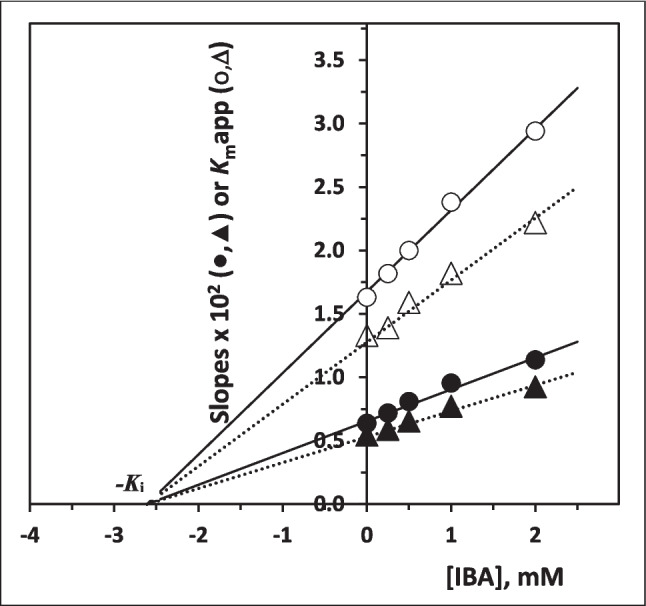
Secondary plots for determination of *K*_i_ values for IBA. Fixed 1 mM [CDNB] and variable [GSH] (solid line) slope (○), and intercept (●); and fixed 1 mM [GSH] and variable [CDNB] (dashed line); slope (Δ), intercept (▲)

Kinetic analysis in the presence of IBA demonstrated competitive inhibition toward both GSH and CDNB (Fig. 6A, B). *K*_i_ values obtained from nonlinear regression analysis were 3.33 ± 0.22 mM for GSH and 2.14 ± 0.16 mM for CDNB. Secondary plot analysis yielded *K*_i_ values of 2.61 mM for both substrate (Fig. 7, Table 1).

### Structural and mechanistic insights into GSTP1-1–auxin interactions

**Fig. 8:**
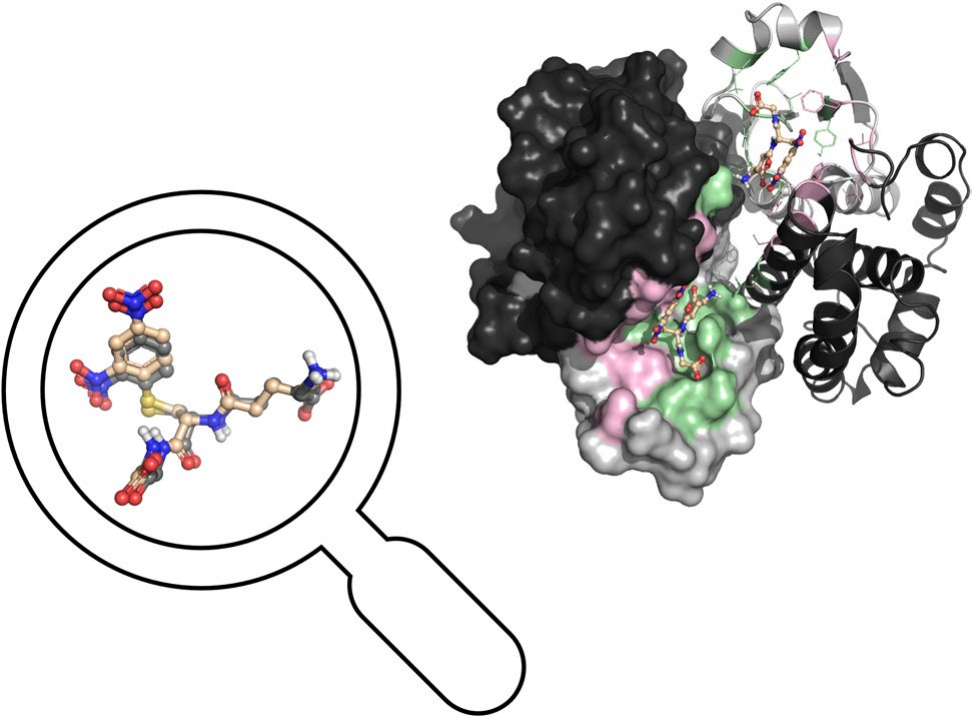
Human GSTP1-1 homodimer with bound GS–DNB (PDB ID: 18GS). Two monomers are shown in surface and cartoon representation, respectively. Each monomer comprises an N-terminal domain (light gray) and a C-terminal domain (dark gray). The N- and C-terminal domains include most of the amino acid residues that form the G-site (green) and the H-site (pink). The native GS–DNB conjugates are shown in ball-and-stick representation. The inset magnifier depicts the superposed structures of native (wheat) and redocked (gray) GS–DNB molecules. The images were rendered using PyMOL Molecular Graphics System, v18 (Schrödinger LLC, Portland, OR, USA)

**Fig. 9:**
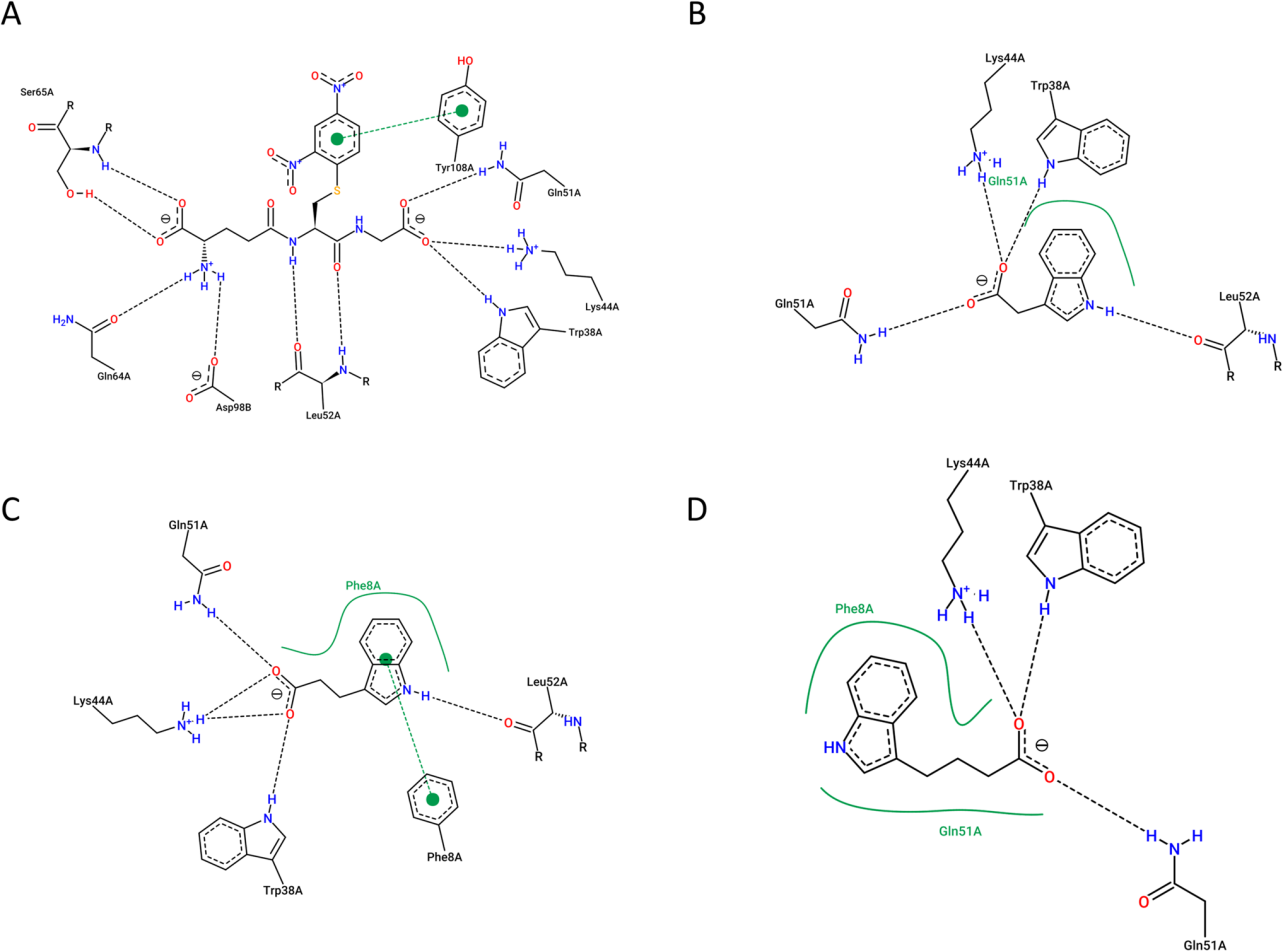
Molecular docking of auxins onto human GSTP1-1. Favorable noncovalent interactions between human GSTP1-1 and (**A**) native GS–DNB, (**B**) cross-docked IAA, (**C**) cross-docked IPA, or (**D**) cross-docked IBA. Black dashed lines represent hydrogen-bonding and electrostatic interactions. Green dashed lines represent aromatic stacking interactions. Green solid lines represent hydrophobic interactions. The interactions were estimated using the PoseView protein–ligand interaction mapping tool (Fricker et al. 2004; Stierand et al. 2006) available at https://proteins.plus

An in silico analysis of human GSTP1 (UniProtKB entry: P09211; PDB entry: 18GS) disclosed that each monomer of the homodimeric enzyme has a total of 209 amino acid residues and is divided into two separate structural domains: the N-terminal domain (residues 1 to 80) folding into a thioredoxin-like topological arrangement of α-helices and β-sheets and the larger C-terminal domain (residues 81 to 209) containing α-helices only (Fig. 8, main). Two functionally distinct subsites make up the active site of the enzyme (one on each monomer): a GSH-binding site (G-site) and a hydrophobic electrophile-binding site (H-site). Tyr7, Trp38, Lys44, Gln51, Leu52, Gln64, Ser65, and Asp98′ (from the opposite monomer) line the G-site; Phe8, Val10, Arg13, Val35, Cys101, Ile104, Tyr108, Asn204, and Gly205 line the H-site (Mohana and Achary 2017). The results of redocking simulations revealed that JAMDA was able to faithfully reproduce the native binding pose of GS–DNB, with a root-mean-square deviation of 0.642 Å and a JAMDA score of − 3.163 (Fig. 8, inset). Cross-docking simulations, on the other hand, showed that IAA, IPA, and IBA could be housed well within the active-site pocket of GSTP1, albeit with comparatively lower JAMDA scores (− 1.445, − 1.483, and − 1.706, respectively). A close look at the favorable noncovalent interactions that occur between GSTP1-1 and GS–DNB indicates that the carboxyl group of the C-terminal glycine residue in GSH structure is electrostatically attracted to the side chain of Lys44 and hydrogen-bonded to the side chains of Trp38 and Gln51, all from the enzyme’s G-site (Fig. 9A). The same trio of amino acids can fulfil an important function in the stabilization of the carboxyl group of IAA, IPA, or IBA (Fig. 9B, C, D). In addition, the main chain of Leu52 (also from the G-site) is in sufficiently close proximity to the N–H proton on the indole ring of IAA or IPA to establish a hydrogen bond. Last, Phe8 from the H-site is able to engage in an aromatic stacking interaction with the indole ring of IPA. Further hydrophobic contacts with these critical residues can help anchor each auxin in the active-site cavity of GSTP1-1.

## Discussion

Cancer is considered one of the leading causes of mortality and is among the noncommunicable diseases responsible for the majority of deaths worldwide. In 2020, the World Health Organization (WHO) ranked cancer as the first or second leading cause of death before the age of 70 in more than half of the countries around the world, and it was also a substantial cause of death in the remaining countries (Sung et al. 2021). Despite significant advances in cancer research, including improvements in treatment, prevention, and diagnosis, cancer remains a major cause of mortality.

Current cancer treatment strategies include surgical removal of cancerous tissue, followed by radiotherapy or the administration of chemotherapeutic agents to eliminate residual malignant cells. Although these interventions have resulted in significant improvements in survival rates and the overall health status of cancer patients, tumors do not always fully regress. The major reasons for cancer therapy failure include detection of the disease and resistance to chemotherapeutic agents used in clinical practice. One of the most common mechanisms of drug resistance is enhanced drug detoxification mediated by detoxifying enzymes such as glutathione S-transferases (GSTs), which can be exploited by malignant cells to increase survival through the acquisition of resistance to anticancer drugs (Coles and Ketterer 1990; Sheehan et al. 2001; Hayes et al. 2005; Alnasser 2025). Drug detoxification is accomplished through several processes involving a variety of enzymes, most notably GSTs. This superfamily of phase II detoxification enzymes neutralizes pesticides, cytotoxic drugs, and genotoxic compounds by catalyzing their conjugation to GSH and consequently forming less toxic/mutagenic glutathione complexes that can readily be eliminated from the body via urinary excretion (Coles and Ketterer 1990; Tew and Gate 2001; Hayes et al. 2005). The successful treatment of metastatic cancers often requires the use of multiple toxic chemotherapeutic agents, as resistance to individual drugs frequently develops. Consequently, cancer biologists have been working to explain the mechanisms behind multidrug resistance (MDR) (Szakacs et al. 2006). Since 1985, the role of GSTs in cancer drug resistance has been well established (Wang and Tew 1985). The overexpression of GSTs and their involvement in the detoxification of anticancer drugs across various cancer tissues have stimulated interest in the identification or synthesis of GST inhibitors or prodrugs capable of preventing the development of resistance. Ethacrynic acid was the first GST inhibitor shown to possess promising inhibitory activity in vitro, however, its clinical use was limited due to adverse effects and restricted isoenzyme specificity (Tew and Gate 2001).

Aside from GST overexpression, which facilitates the detoxification of chemotherapeutic agents, multiple studies have shown a synergistic involvement of both GSTs and efflux pumps in drug resistance (Meijerman et al. 2008; Sau et al. 2010). Concurrently, anticancer drug detoxification via GSTs is highly connected to the efflux transporter-assisted export of detoxified compounds. The resulting glutathione conjugates are eliminated from the cells by efflux transporters such as P-glycoprotein and multidrug resistance-associated protein (MRP1), both members of the ATP-binding cassette (ABC) transporter superfamily (Keppler 1999; Meijerman et al. 2008). Collectively, these findings suggest that MDR develops through synergistic mechanisms in cancer cells. In addition to their catalytic functions, GSTs also contribute to MDR through noncatalytic activities, including modulation of the c-Jun N-terminal kinase (JNK) signaling pathway. Inhibition of this pathway by GSTs protects cancer cells from apoptosis, thereby promoting cell survival (Allocati et al. 2018).

Researchers have been identifying and developing various types of inhibitors capable of blocking GST activity in an effort to overcome the aforementioned limitation. Among these inhibitors, several plant-derived compounds have been recognized as relatively potent inhibitors. A study examining plant-derived flavonoids and polyphenols-namely 2-hydroxychalcone, morin, tannic acid, quercetin, and butein-demonstrated that these compounds exhibited varying degrees of inhibitory potency against rat liver GST. Tannic acid identified as the most potent inhibitor, with an *IC*_50_ value of 1.044 μM. It inhibited rat liver GST in a competitive manner with respect to GSH and exhibited noncompetitive inhibition toward CDNB. 2-Hydroxylchalcone ranked second, with an *IC*_50_ value of 6.76 μM. Butein, morin, and quercetin also inhibited rat liver GST with *IC*_50_ values of 9.03, 13.71, and 18.73 μM, respectively, placing them below tannic acid and 2-hydroxychalcone in terms of inhibitory potency (Zhang and Das 1994).

The inhibitory effect of quercetin on human GSTP1-1 was also studied in a separate study, which demonstrated that quercetin inhibits human GSTP1-1 in a time- and concentration-dependent manner. Quercetin is a naturally occurring polyphenol and a prominent constituent of vegetables, fruits, red wine, nuts, and tea. Incubation of GSTP1-1 with quercetin at concentrations of 1 and 10 μM for 2 h resulted in reductions in enzymatic activity of 25% and 42%, respectively (Van Zanden et al. 2003).

Furthermore, curcumin and ellagic acid, two naturally occurring plant-derived compounds, were shown to inhibit the activity of GSTP1-1 in a concentration- and time-dependent manner, with *IC*_50_ values ranging from 0.04 to 5 μM, curcumin being the more potent inhibitor. Both compounds primarily exhibited mixed-type inhibition and to a lesser extent, uncompetitive inhibition at the H- and G-sites of the enzyme (Hayeshi et al. 2007). These findings highlight the inhibitory potential of plant-derived compounds against human GSTs and underscore the importance of investigating phytohormones as possible modulators of *hp*GSTP1-1 activity. Human pi-class glutathione *S*-transferases represent a particularly important GST subclass, as they serve as promising targets for the discovery or synthesis of inhibitors that may enhance chemotherapeutic efficacy and help overcome MDR in patients with metastatic cancer (Van Zanden et al. 2003).

It was discovered that auxins can bind to plant GSTs, leading to the elucidation of the functional significance of this interaction. Two distinct roles of auxin binding to GSTs have been proposed. First, auxins may bind to GSTs as substrates, resulting in the formation of a glutathionyl (GS–) conjugate. Second, auxins may bind as non-substrate ligands. The formation of this conjugate has been demonstrated to regulate GST activity accordingly (Bilang et al. 1993; Zettl et al. 1994). In the first scenario, the binding of IAA to plant GSTs as a substrate leads to its metabolism and influences intracellular auxin levels. However, when the auxins interact with GSTs as ligands, they induce changes in cellular glutathione content and alter cellular redox state, which may in turn affect various biological processes (Bilang et al. 1993).

To evaluate the physiological importance of auxin binding to plant GSTs, the potential inhibitory effects of auxins were examined using different GST isoenzymes expressed in *Escherichia coli.* For example, recombinantly produced GST5 isoenzyme from *Arabidopsis* exhibited *K*_m_ values of 0.86 and 1.29 mM for GSH and CDNB, respectively. The same study further demonstrated that IAA, 2,4-dichlorophenoxyacetic acid (2,4-D), 1-naphthaleneacetic acid (1-NAA) and 2-NAA inhibited the GST5 activity competitively with respect to GSH, with IAA having a *K*_i_ value of 1.56 mM (Watahiki et al. 1995).

Collectively, these findings from previous studies underscore the importance of conducting an in-depth investigation into the inhibitory effects of auxins on GSTs not only in plants but also in other organisms, including humans. In the present study, three auxins—indole-3-acetic acid (IAA), indole-3-propionic acid (IPA), and indole-3-butyric acid (IBA)—were examined. All endogenous (naturally occurring) auxins possess an aromatic fused heterocycle and a carboxylic acid group, with IAA being the most prominent auxin and exerting substantial effects at both local and whole-plant levels. However, despite its biological prominence, IAA was the least potent inhibitor of *hp*GSTP1-1 activity among the tested auxins. Across the five tested concentrations (0.3125–5 mM), IAA exhibited an *IC*_50_ value of 7.9 mM.

IAA exhibited *K*_*i*_ values of 4.00 ± 0.62 mM (statistical analysis)/2.38 mM (secondary plots) at fixed CDNB and variable GSH concentrations ([CDNB]_f_–[GSH]_v_), and 3.30 ± 0.24 mM (statistical analysis)/2.38 mM (secondary plots) at fixed GSH and variable CDNB concentrations ([GSH]_f_–[CDNB]_v_). IPA was a more potent inhibitor of *hp*GSTP1-1 than IAA, with an *IC*_50_ value of 6.5 mM. The *K*_*i*_ values for IPA at [CDNB]_f_–[GSH]_v_ and [GSH]_f_–[CDNB]_v_ were 3.33 ± 0.23 mM (statistical analysis)/2.84 mM (secondary plots) and 3.52 ± 0.24 mM (statistical analysis)/2.84 mM (secondary plots), respectively. The most potent auxin in inhibiting the activity of *hp*GSTP1-1 was IBA, with an *IC*_50_ value of 4.2 mM. The *K*_i_ values for IBA at [CDNB]_f_–[GSH]_v_ and [GSH]_f_–[CDNB]_v_ from statistical analysis/the secondary plot were 3.33 ± 0.22 mM/2.61 mM and 2.14 ± 0.16 mM/2.61 mM, respectively. All three auxins inhibited *hp*GSTP1-1 in a competitive mechanism with respect to both GSH and CDNB.

The definitive *K*_i_ values reported in this study are obtained from nonlinear regression analysis, where kinetic parameters are calculated using all substrate and inhibitor concentration data simultaneously. This cumulative statistical approach provides a more robust estimation of kinetic parameters. Secondary plots, on the other hand, are constructed by evaluating substrate concentration series separately for each inhibitor concentration. These plots are presented for illustrative and mechanistic validation purposes, allowing visualization of inhibition patterns and consistency with classical enzyme kinetics. Importantly, when secondary plots are used, the influence of measurement errors and distortions introduced by Lineweaver–Burk linearization is reduced compared to relying solely on reciprocal plots.

Interpretation of the present findings requires consideration of the concentrations of auxins that are encountered (endogenously and/or by diet) in human biological systems relative to those used in vitro. Although classical plant auxins such as indole-3-acetic acid (IAA) have been detected in mammalian systems as a result of gut microbial metabolism and, systemic concentrations of IAA in humans are generally in the low micromolar range, far below the millimolar concentrations used in our inhibition assays (Dou et al., 2015). Moreover, other indole derivatives related to plant auxins—including indole-3-propionic acid (IPA), indole-3-butyric acid (IBA), and various gut microbial metabolites—are also detectable in human plasma as products of dietary tryptophan metabolism by microbiota, but again typically at micromolar or sub-micromolar concentrations (Anderson 2023). Dietary intake of auxin-like compounds from plant foods contributes additional exposure, but these sources generally deliver trace amounts that are rapidly metabolized or modified by gut microbiota. Collectively, these observations underscore that, while testing high concentrations in vitro is useful for characterizing biochemical interaction potential, the direct physiological relevance of GSTP1-1 inhibition by auxins at typical human exposures remains low.

The computationally predicted interactions and complementarity between human GSTP1-1 and the auxins in question were consistent with the in vitro observation that IBA exhibited the highest inhibitory effect among the tested compounds (JAMDA score: − 1.706), while remaining a weak inhibitor overall.

Although IAA, IPA, and IBA were able to engage in favorable noncovalent interactions (primarily hydrogen-bonding and electrostatic interactions) with key amino acid residues from the G- and H-subsites of *hp*GSTP1-1, these interactions were limited in extent and are indicative of low-affinity binding. Within this context, IBA may act as a slightly more effective occupier of the active-site cavity due to its comparatively longer alkanoic acid side chain, providing a structural rationale for its modestly greater inhibitory potency rather than implying enhanced pharmacological relevance. The molecular docking results are therefore explicitly framed as qualitative and hypothesis-supporting, and not as predictors of inhibitory potency or translational relevance.

## Conclusion

In conclusion, the present work provides a comprehensive mechanistic characterization of the interaction between human placental GSTP1-1 and three structurally related auxins, namely indole-3-acetic acid, indole-3-propionic acid, and indole-3-butyric acid. Through combined enzyme kinetic and molecular docking analyses, these auxins were shown to inhibit *hp*GSTP1-1 activity in a competitive manner with respect to both glutathione and CDNB, albeit with low affinity and *IC*_50/_*K*ᵢ values in the millimolar range. The weak inhibitory potency observed indicates that these compounds are unlikely to exert meaningful *hp*GSTP1-1 modulation under normal physiological conditions. However, the consistency between kinetic behavior and docking-based structural predictions highlights that auxins can occupy the active-site cavity of *hp*GSTP1-1 and engage key residues within the G- and H-sites. Among the tested compounds, IBA exhibited slightly stronger inhibition, which may be attributed to its longer alkanoic side chain and improved accommodation within the active site.

Overall, this study should be regarded as a mechanistic in vitro*/*in silico investigation rather than a demonstration of immediate pharmacological applicability. The findings contribute to a broader understanding of how indole-based small molecules interact with *hp*GSTP1-1 and may serve as a starting point for future studies exploring structurally optimized derivatives or other indole-containing metabolites with enhanced inhibitory potential.

## Limitations

A key limitation of the present study is the relatively weak inhibitory potency of the tested auxins toward *hp*GSTP1-1, as evidenced by *IC*₅₀ and *K*ᵢ values in the low-millimolar range. Such concentrations are substantially higher than those typically encountered under normal physiological conditions, which limits the direct translational or pharmacological relevance of these findings. Accordingly, the observed inhibition should be interpreted as a biochemical interaction demonstrated under controlled in vitro conditions rather than as evidence of meaningful enzyme modulation in vivo. While molecular docking provides structural insight into potential binding modes, further studies incorporating physiologically relevant concentrations, and in vivo models would be required to clarify whether auxin–GSTP1-1 interactions have any functional relevance in human biology.

## Data Availability

Data available on request from the authors.
